# Human Infection with *Candidatus* Neoehrlichia mikurensis, China

**DOI:** 10.3201/eid1810.120594

**Published:** 2012-10

**Authors:** Hao Li, Jia-Fu Jiang, Wei Liu, Yuan-Chun Zheng, Qiu-Bo Huo, Kun Tang, Shuang-Yan Zuo, Kun Liu, Bao-Gui Jiang, Hong Yang, Wu-Chun Cao

**Affiliations:** Beijing Institute of Microbiology and Epidemiology, Beijing, People’s Republic of China (H. Li, J.-F. Jiang, W. Liu, K. Tang, S.-Y. Zuo, K. Liu, B.-G. Jiang, H. Yang, W.-C. Cao);; and Mudanjiang Forestry Central Hospital, Mudanjiang, People’s Republic of China (Y.-C. Zheng, Q.-B. Huo)

**Keywords:** *Candidatus* Neoehrlichia mikurensis, bacteria, human infection, ticks, rodents, vector-borne infections, China

## Abstract

To identify *Candidatus* Neoehrlichia mikurensis infection in northeastern China, we tested blood samples from 622 febrile patients. We identified in 7 infected patients and natural foci for this bacterium. Field surveys showed that 1.6% of ticks and 3.8% of rodents collected from residences of patients were also infected.

*Candidatus* Neoehrlichia mikurensis was detected in 1999 in *Ixodes ricinus* ticks in the Netherlands and referred to as an *Ehrlichia* spp.–like agent (*1*). It was then classified as a new member of family *Anaplasmataceae* on the basis of ultrastructure and phylogenetic analysis (*2*). The agent was detected in ticks and small wild mammals in Europe and Asia (*1*–*6*) and has recently been reported to infect humans, especially immunocompromised patients in Europe (*7*–*10*). However, no cases of infection have been identified outside Europe. Moreover, the agent has not yet been isolated in pure culture, and its antigens are not available.

To investigate human infections with tick-borne agents in China, we initiated a surveillance study at Mudanjiang Forestry Central Hospital (Mudanjiang, China). This hospital is one of the largest hospitals treating patients with tick-borne infectious diseases in northeastern China, where various tick-borne agents have been detected in ticks and animal hosts (*11*–*15*).

## The Study

**Figure 1:**
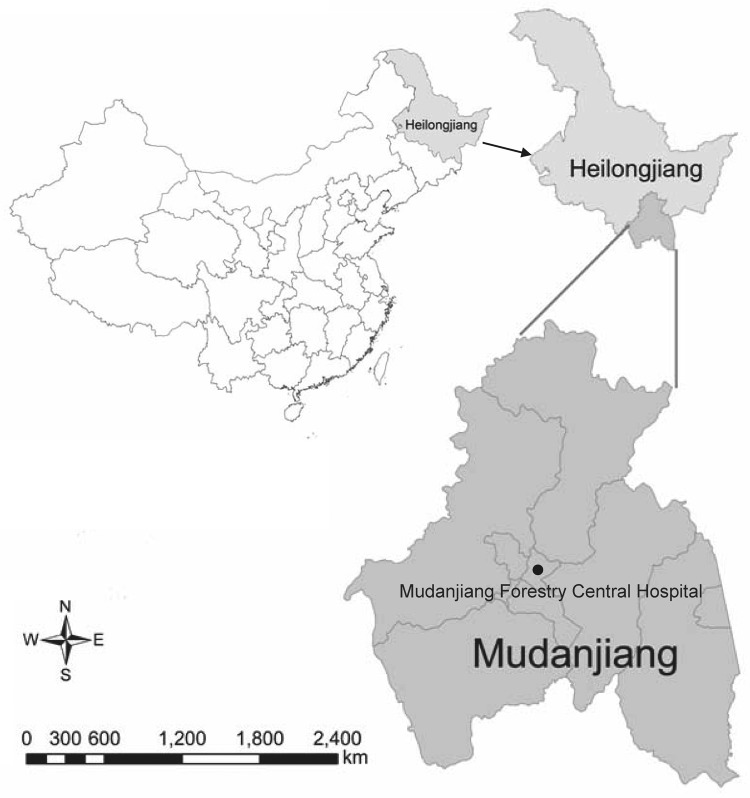
Location of Mudanjiang, Heilongjiang Province, China, where *Candidatus* Neoehrlichia mikurensis was detected.

During May 2–July 30, 2010, a total of 622 febrile patients, who had histories of recent tick bites and sought treatment at Mudanjiang Forestry Central Hospital (Figure 1) were screened for the infections of tick-borne agents. When patients were admitted, peripheral blood samples were collected and treated with EDTA. DNA as extracted by using the QIAmp DNA Blood Mini Kit (QIAGEN, Germantown, MD, USA).

For a broad-range assay, a nested PCR specific for the 16S rRNA gene (*rrs*) was used to detect organisms in the family *Anaplasmataceae*. For positive samples, 2 heminested PCRs were used to amplify the entire *rrs* gene. For further confirmation, a nested PCR specific for the 60-kDa heat shock protein gene (*groEL*) was performed. Detailed cycling conditions for all amplifications are described in the Technical Appendix.

Seven patients were found to be infected with *Candidatus* N. mikurensis by amplifications of the *rrs* and *groEL* genes. Amplified *rrs* gene (1,501 bp) and partial *groEL* gene (1,230 bp) sequences from these patients were identical. These sequences were also identical to genes of *Candidatus* N. mikurensis detected in ticks and rodents in the Asian region of Russia (*5*).

Serum samples were collected from patients during the acute (2–12 days after onset of illness) or convalescent (34–42 days after onset of illness) phases of illness. All samples were negative for IgG against *Anaplasma phagocytophilum*, *Ehrlichia chaffeensis*, *Borrelia burgdorferi*, *Rickettsia heilongjiangensis*, and tick-borne encephalitis virus when tested by indirect immunofluorescence assay.

All 7 patients were farmers residing in the villages in Mudanjiang. Their median age was 41 years (range 29–67 years) and 5 were men. None had been vaccinated against tick-borne encephalitis. The patients had onset of illness during May 20–July 13, 2010. The median time from the tick bite to the onset of illness and from the onset of illness to the physician visit was 8 days (range 2–35 days) and 7 days (range 1–12 days), respectively.

All patients were otherwise healthy, and none had a history of underlying immunocompromised conditions. Fever, headache, and malaise were reported for all 7 patients. Other major manifestations included nausea (5/7), vomiting (5/7), myalgia (4/7), and stiff neck (4/7). Less common symptoms were arthralgias (2/7), cough (2/7), diarrhea (1/7), confusion (1/7), and erythema (1/7). Skin erythema (multiple and oval) was seen on the neck of 1 patient.

Laboratory test results showed leukopenia in 1 patient, leukocytosis in 1 patient, thrombocytopenia in 2 patients, and anemia in 2 patients. Serum levels of alanine aminotransferase and aspartate aminotransferase were within reference ranges for all patients. Wright–Giemsa stained peripheral blood smears did not show morulae or other blood parasites.

To identify local natural foci, we performed a field investigation on infections of *Candidatus* N. mikurensis in ticks and rodents from areas of residences of the patients. During May–July 2010, a total of 516 host-seeking ticks, including 316 *I. persulcatus*, 187 *Haemaphysalis concinna*, and 13 *Dermacentor silvarum*, were collected on vegetation and individually examined. *Candidatus* N. mikurensis DNA was detected in 6 (1.9%) *I. persulcatus* and 2 (0.8%) *H. concinna* ticks, but no DNA was detected in *D. silvarum* ticks (Table).

**Table:** Prevalence of *Candidatus* Neoehrlichia mikurensis in ticks and rodents, Mudanjiang, China

Species	No. positive/no. tested (%)
Tick	
* Ixodes persulcatus*	6/316 (1.9)
* Haemaphysalis concinna*	2/187 (0.8)
* Dermacentor silvarum*	0/13 (0)
Total	8/516 (1.6)
Rodent	
* Clethrionomys rufocanus*	5/109 (4.6)
* Rattus norvegicus*	2/35 (5.7)
* Apodemus peninsulae*	0/30 (0)
* Apodemus agrarius*	0/25 (0)
* Mus musculus*	0/9 (0)
* Tamias sibiricus*	1/3 (33.3)
Total	8/211 (3.8)

A total of 211 rodents of various species were captured by using snap traps. After rodent species was identified, spleen specimens were collected for DNA extraction and PCR. Eight rodents of 3 species, 5 (4.6%) *Clethrionomys rufocanus*, 2 (5.7%) *Rattus norvegicus*, and 1 (33.3%) *Tamias sibiricus*, were positive for *Candidatus* N. mikurensis (Table).

**Figure 2:**
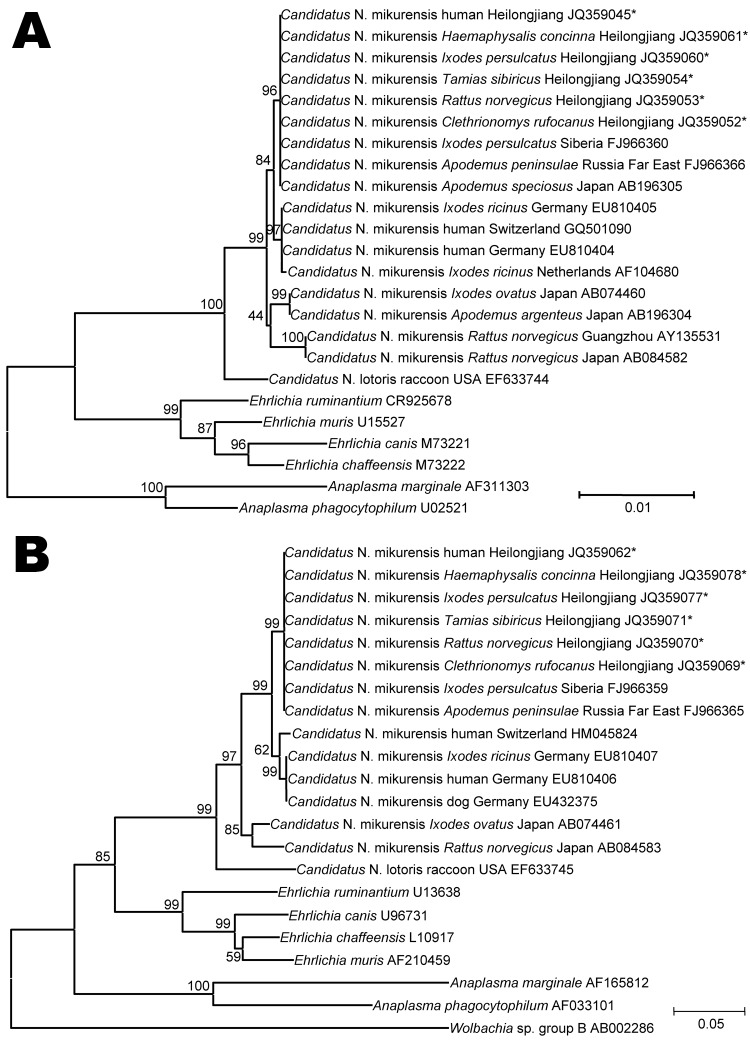
A) Neighbor-joining trees based on the 16S rRNA gene (*rrs*) and B) the 60-kDa heat shock protein gene (*groEL*) of *Candidatus* Neoehrlichia mikurensis, China, generated by using Molecular Evolutionary Genetics Analysis software version 4.0, (www.megasoftware.net/) the maximum composite-likelihood method, and bootstrap analysis of 1,000 replicates. Asterisks indicate nucleotide sequences of *Candidatus* N. mikurensis determined in this study. Numbers on branches indicate percentage of replicates that reproduced the topology for each clade. Scale bars indicate estimated evolutionary distance. A total of 1,303 positions for *rrs* and 953 positions for *groEL* were analyzed. Sources of *Candidatus* N. mikurensis sequences are shown between species names and GenBank accession numbers.

Nucleotide sequences of *rrs* and *groEL* genes of 8 ticks and 8 rodents were identical to each other and to sequences obtained from the 7 patients. Phylogenetic analysis of *rrs* genes showed that nucleotide sequences identified were identical to those of *Candidatus* N. mikurensis from Japan and the Asian region of Russia but different from sequences from Europe (99.6%–99.8% similarity) (Figure 2, panel A). Similar phylogenetic relationships were observed in a neighbor-joining tree based on *groEL* gene nucleotide sequences. In comparison with sequences from humans and ticks in Europe, the *groEL* gene sequences identified in the study showed 97.6%–98.4% similarity (Figure 2, panel B).

## Conclusions

We have detected *Candidatus* N. mikurensis DNA in blood samples from 7 patients collected during the period of acute illness, which suggests that this bacterium was the etiologic agent of the infections. Our findings demonstrated human infections with *Candidatus* N. mikurensis in China. The *rrs* and *groEL* gene nucleotide sequences of this *Candidatus* N. mikurensis variant were identical to those obtained from ticks and rodents in the Asian region of Russia, which have not been reported to cause human infection.

Unlike reported cases in elderly or immunocompromised patients in whom disease developed (*7*–*10*), all 7 patients in our study had relatively mild disease. Major clinical manifestations and laboratory findings of the cases in our report, such as leukocytosis, were not similar to those of previously reported cases. It is noteworthy that the patients reported in this study were previously healthy. Thus, their clinical manifestations might be typical of *Candidatus* N. mikurensis infection in an otherwise healthy population. However, the number of cases in our study was limited, and clinical data were not inclusive. Clinical characteristics of *Candidatus* N. mikurensis infection should include detailed descriptions of additional cases.

Our finding of a *Candidatus* N. mikurensis variant in 1.6% of ticks and 3.8% of rodents tested suggested natural foci of the bacterium in Mudanjiang. Therefore, clinical diagnosis of *Candidatus* N. mikurensis infection should be considered in patients who have been exposed to areas with high rates of tick activity. It is noteworthy that *Candidatus* N. mikurensis was originally detected in *R*. *norvegicus* from Guangzhou Province in southeastern China (*4*), thereby indicating the potential threat to humans in areas other than northeastern China.

In summary, we identified *Candidatus* N. mikurensis as an emerging human pathogen in China. Further studies should be conducted to isolate this bacterium and investigate its epidemiologic, genetic, and pathogenic features. To guide diagnostic testing and treatment, physicians should be aware that human *Candidatus* N. mikurensis infections are in Heilongjiang Province and that PCR can be used as a diagnostic technique for identifying suspected infections.

Technical Appendix PCR, morphologic, and serologic procedures used for detection of *Candidatus* Neoehrlichia mikurensis, Mudanjiang, China.
