# Supplementary material for: Human Infection with Candidatus Neoehrlichia mikurensis, China
Source: Emerg Infect Dis. 2012 Oct;18(10):1636–9. doi: 10.3201/eid1810.120594 (PMC3471638; doi:10.3201/eid1810.120594)
Supplement: Technical Appendix — PCR, morphologic, and serologic procedures used for detection of Candidatus Neoehrlichia mikurensis, Mudanjiang, China. [file 12-0594-Techapp-s1.pdf]

Article DOI: http://dx.doi.org/10.3201/eid1810.120594

Human Infection with *Candidatus* Neoehrlichia mikurensis, China

Technical Appendix

PCR, morphologic, and serologic procedures used for detection of *Candidatus* Neoehrlichia mikurensis, Mudanjiang, China

PCR

For broad-range assay, a nested PCR specific for the 16S rRNA (*rrs*) gene was used to detect all known species of the family *Anaplasmataceae* (Technical Appendix Table). PCR amplifications were performed in a 30-µL reaction volume in GeneAmp PCR System 9700 (Applied Biosystems, Foster City, CA, USA).

For initial amplification, the reaction mixture contained 0.8 µmol/L each of primers Eh-out1 (*1*) and 3–17U, 200 mmol/L of each dNTP, 1 unit of Taq polymerase, 3 µL of 1× PCR buffer, and 3 µL of purified DNA. Cycling conditions were an initial 5-min denaturation at 94°C; 40 cycles at 94°C for 40 s, 55°C for 40 s, and 72°C for 1 min 45 s; and a final extension at 72°C for 7 min.

For nested amplification, the components were similar to those used in the initial amplification, except that 0.5 µmol/L of EHR16SD and 0.5 µmol/L of EHR16SR (*2*) were used as primers and 1 µL of the primary PCR product was used as template. Cycling conditions were 94°C for 5 min; 35 cycles at 94°C for 30 s, 50°C for 30 s, and 72°C for 30 s; and a final at 72°C extension for 7 min. Nested amplicons were directly sequenced by using primers EHR16SD and EHR16SR.

For positive samples, 2 heminested PCRs were performed to amplify the entire *rrs* gene. Components and conditions in the 2 PCRs were similar to those in the nested PCR, except that

primers Eh-out1 and Eh-out2U were used to amplify 5′-end fragments, and primers Eh-out2fU and 3–17U were used to amplify 3′-end fragments. Amplified 5′-end fragments were sequenced by using primer Eh-out2U, and amplified 3′-end fragments were sequenced by using primers Eh-out2fU and CNM1050f.

For confirmation of identification of *Candidatus* Neoehrlichia mikurensis, a nested PCR specific for the 60-kDa heat shock protein (*groEL*) gene was performed. Components in the nested PCR were the same as those used in amplification of the *rrs* gene. Primers HS3-f and HSVR (*3*) was used for the initial amplification. Primers groEL-2f and groEL-2r were used for nested amplification. Cycling conditions were 94°C for 5 min; 35 cycles at 94°C for 30 s, 55°C for 30 s, and 72°C for 1 min 30s; and a final extension at 72°C for 7 min. Nested amplicons were sequenced by using primers groEL-Sf and groEL-Sr.

All positive amplicons were purified by using E.Z.N.A Gel Extraction Kit (Omega Bio-Tek, Norcross, GA, USA). These amplicons were then sequenced by using an automated DNA sequencer (3730 DNA Sequencer; Applied Biosystems).

To minimize risk for contamination, template isolation and PCR were performed by using specified pipettor sets in separate rooms. Certified DNA/RNase–free filter barrier tips were used to prevent aerosol contamination. All PCRs were performed with appropriate controls.

Morphologic Examination of Peripheral Blood Smears

Fresh peripheral blood smears from patients with PCR-confirmed *Candidatus* Neoehrlichia mikurensis infection were stained with Wright–Giemsa (BaSO Diagnostics, Inc., Zhuhai, China) and examined with a light microscope (BX43; Olympus, Center Valley, PA, USA) for intracellular morulae.

Serologic Testing

Serum samples from patients with PCR-confirmed *Candidatus* Neoehrlichia mikurensis infection were tested by using an indirect immunofluorescence assay for IgG against *Anaplasma*

phagocytophilum (*4*), *Ehrlichia chaffeensis* (*Ehrlichia chaffeensis* IFA IgG Substrate Slide; Focus Diagnostics, Inc., Cypress, CA, USA), *Borrelia burgdorferi* (established in our laboratory), tick-borne encephalitis virus (*5*), and *Rickettsia heilongjiangensis* (*6*).

References

1. Wen B, Jian R, Zhang Y, Chen R. Simultaneous detection of *Anaplasma marginale* and a new *Ehrlichi*a species closely related to *Ehrlichia chaffeensis* by sequence analyses of 16S ribosomal DNA in *Boophilus microplus* ticks from Tibet. J Clin Microbiol. 2002;40:3286–90. PubMed http://dx.doi.org/10.1128/JCM.40.9.3286-3290.2002

2. Parola P, Roux V, Camicas JL, Baradji I, Brouqui P, Raoult D. Detection of *ehrlichiae* in African ticks by polymerase chain reaction. Trans R Soc Trop Med Hyg. 2000;94:707–8. PubMed http://dx.doi.org/10.1016/S0035-9203(00)90243-8

3. Liz JS, Anderes L, Sumner JW, Massung RF, Gern L, Rutti B, et al. PCR detection of granulocytic ehrlichiaein *Ixodes ricinus* ticks and wild small mammals in western Switzerland. J Clin Microbiol. 2000;38:1002–7. PubMed

4. Zhan L, Cao WC, Jiang JF, Zhang XA, Liu YX, Wu XM, et al. *Anaplasma phagocytophilum* from rodents and sheep, China. Emerg Infect Dis. 2010;16:764–8. PubMed

5. Li ZT. Investigation of indirect immunofluorescence assay in the early diagnosis of tick-borne encephalitis patients [in Chinese]. Chinese Journal of Preventive Medicine. 1985;19:85–7.

6. Duan C, Meng Y, Wang X, Xiong X, Wen B. Exploratory study on pathogenesis of far-eastern spotted fever. Am J Trop Med Hyg. 2011;85:504–9. PubMed http://dx.doi.org/10.4269/ajtmh.2011.10-0660

Technical Appendix Table. Nucleotide sequences of primers used for detection of *Candidatus* Neoehrlichia mikurensis by PCR, China*

Gene	Primer	Sequence (5′→3′)	Reference

Technical Appendix Table. Nucleotide sequences of primers used for detection of *Candidatus* Neoehrlichia mikurensis by PCR, China*			
rrs	Eh-out1 (ap)	TTGAGAGTTTGATCCTGGCTCAGAACG	(*1*)
	Eh-out2U (ap, s)	CACCTCTACACTAGGAATTCCACTATC	(*1*) modified
	3–17U (ap)	WAAGGWGGTAATCCAGC	(*1*) modified
	EHR16SD (ap, s)	GGTACCYACAGAAGAAGTCC	(*2*)
	EHR16SR (ap, s)	TAGCACTCATCGTTTACAGC	(*2*)
	Eh-out2fU (ap, s)	GATAGTGGAATTCCTAGTGTAGAGGTG	(*1*) modified
	CNM1050f (s)	TAACCCTTGTCCTTAGTTGCC	This study
groEL	HS3-f (ap)	ATAGTYATGAAGGAGAGTGAT	(*3*)
	HSVR (ap)	TCAACAGCAGCTCTAGTWG	(*3*)
	groEL-2f (ap)	AAAGTTTAAGAGTTCGCCTC	This study
	groEL-2r (ap)	TCTACTTCGCTTGAACCACC	This study
	groEL-Sf (s)	TACAGTTGAAGAAAGTAAGGG	This study
	groEL-Sr (s)	CAAAATAAGGCGATAGATAACC	This study

**rrs,* 16S rRNA; ap, amplification primer; s, sequencing primer; *groEL*, 60-kDa heat shock protein.
